# A roadmap to medical nutrition therapy in type 2 diabetes

**DOI:** 10.1007/s42000-023-00483-1

**Published:** 2023-09-21

**Authors:** Almog Shalit, Stavroula A. Paschou, Theodora Psaltopoulou

**Affiliations:** Endocrine Unit and Diabetes Center, Department of Clinical Therapeutics, School of Medicine, Alexandra Hospital, National and Kapodistrian University of Athens, 80 Vasilisis Sophias, 11528 Athens, Greece

Type 2 diabetes mellitus (T2DM) is a major global health problem with tremendous impact on economic and social aspects as well. In 2021, the International Diabetes Federation estimated that 536 million adults were living with T2DM worldwide and predicted an exponential increase in prevalence in the coming years [1]. Its epidemic proportions and devastating consequences call for effective treatment interventions. The three pillars of T2DM management include lifestyle modifications, pharmacotherapy, and surgery. Specifically, medical nutrition therapy (MNT) is an affordable and accessible option with strong evidence supporting its efficacy in the prevention, regulation, and even reversal of the disease [2].

Given the tight association between obesity and T2DM, sustained weight loss is integral to MNT, the aim being for an initial 5–10% loss with greater loss denoting even greater benefit [3]. The intervention usually consists of 500–750 kcal daily deficit, resulting in nutrition plans of approximately 1200–1500 kcal/day for women and 1500–1800 kcal/day for men. Additionally, in carefully selected individuals, very-low-calorie diets (800–1000 kcal/day) with total meal replacements can be attempted for 12–20 weeks in order to achieve significant initial results. The principal goal is to maintain the achieved loss [3].

Although weight loss guidelines are well established, the same cannot be said for the nutritional plan [4]. The general consensus advises incorporating non-starchy vegetables, legumes, fresh fruits, whole grains, and low-fat dairy products while limiting processed foods, refined carbohydrates, added sugars, and trans-fat [5]. Useful meal planning strategies include the T2DM plate method and carbohydrate counting. However, research to establish an ideal dietary composition is still inconclusive [4]. A growing database indicates that in a hypocaloric diet plan, there is no optimal macronutrient distribution and recommendations should be tailored to personal preferences in order to ensure long-term adherence [4]. Further individualization might be feasible in the future through nutrigenomics and metabolomics [5].

Reducing the overall carbohydrate consumption by adopting low- or very-low-carbohydrate dietary patterns (≤ 10 and 10–26%, respectively) has proven effective in blood glucose regulation and weight loss trials. However, it is arguable whether such dietary modifications can be sustained long term [4]. Additional concerns have been voiced over a compensatory increase in dietary fat, including saturated fat, possibly increasing cardiovascular risk [2]. Lastly, very-low-carbohydrate eating patterns are not recommended during pregnancy or breastfeeding or for people with eating disorders and those with renal dysfunction. Relative caution should also be employed with concomitant use of sodium glucose cotransporter 2 inhibitors (SGLT-2i) [4]. Notably, the utility of the glycemic index is still uncertain as recent data have disputed the previously claimed benefits of low glycemic index foods [6, 7]. Taking the above into consideration, a moderate carbohydrate intake is usually recommended, focusing on nutrient-dense ingredients [5].

Regular fiber intake is correlated with glycemic regulation and lower overall mortality. Recommendations for people with T2DM aim for at least 14 g/1000 kcal or 25 g/day for women and 38 g/day for men, which is consistent with the guidelines for the general population [8]. It is advised that at least 30% of dietary fiber be soluble and more than half of consumed grains should be whole grains. The importance of additional fiber properties, such as fermentability and viscosity, is also being examined [5].

Protein recommendations for T2DM align with that advised for the general population, amounting to 1.0–1.2 g/kg body weight or corrected body weight for patients with overweight/obesity. Patients with diabetic nephropathy should limit their consumption to 0.8 g/kg [7], but not less [4]. Additional attention is being given to protein source and quality, with the accent on plant-based proteins [7].

Regarding dietary fat, trans fat should be avoided, while saturated fatty acids (SFAs) should be limited to less than 7–9% of total daily energy intake. Alternatively, fat sources should include monounsaturated fatty acids (MUFAs), polyunsaturated fatty acids (PUFAs), and mixed omega-3/omega-6 fatty acids, preferably from plant sources. Practical guidelines include three weekly servings of oily fish, nuts, seeds, and low-fat dairy [5, 7].

Other miscellaneous guidelines regard micronutrients, alcohol, and the use of sweeteners. The routine use of supplementary vitamins (e.g., vitamin D, niacin) inositol, minerals (e.g., zinc and chromium), herbs, or spices (e.g., cinnamon and curcumin) is not recommended but can be considered on an individual basis for underlying deficiencies [5]. Furthermore, the general guidelines for alcohol consumption apply in the case of T2DM (no more than 1 serving/day for women and 2 servings/day for men); however, patients should be educated on the associated risks for weight gain and hypoglycemia [4]. Finally, nonnutritive sweeteners can assist in the weight loss process by reducing the overall calorie and carbohydrate intake, although no glycemic benefit is noted. Guidelines suggest they can be used, though sparingly [4].

Another important aspect of nutritional consultation is meal timing and alignment with the circadian clock. Substantial evidence has demonstrated attenuated glucose regulation and insulin sensitivity at night compared to earlier hours of the day, regardless of the total energy intake [9]. Associations have also been drawn between skipping breakfast and significantly higher overall energy intake, increased postprandial glycemia, and cholesterol values. Consequently, dietary plans should aim for higher energy and carbohydrate proportions in the morning and progressive reduction throughout the day [10].

It is imperative to note that the sequence of food consumption during a meal affects glycemic response. The scientific literature reveals that the consumption of dietary fiber, protein, and lipids before carbohydrates as “preloads” can markedly improve glycemic tolerance [11]. Possible explanations include delayed gastric emptying, increased satiety, and enhanced insulin secretion. Utilizing this strategy achieves better adherence than traditional diets and can be a useful tool in MNT [7].

In conclusion, MNT plays an integral part in T2DM management. Emphasis should be placed on weight loss and the quality of nutritional sources. In contrast, recommendations regarding precise macronutrient patterns are still inconclusive. Active participation of the patient and individual preferences are pivotal during nutritional planning in order to ensure adherence and long-term benefit. Lastly, additional factors worth considering are the timing of meals and the sequence of food consumption during a meal.
